# Nutripedia: The Fight against the Fake News in Nutrition during Pregnancy and Early Life

**DOI:** 10.3390/nu13092998

**Published:** 2021-08-28

**Authors:** Elvira Verduci, Sara Vizzuso, Armanda Frassinetti, Lisa Mariotti, Alberico Del Torto, Giulia Fiore, Annamaria Marconi, Gian Vincenzo Zuccotti

**Affiliations:** 1 Department of Pediatrics, “Vittore Buzzi” Children’s Hospital, 20154 Milan, Italy; sara.vizzuso@asst-fbf-sacco.it (S.V.); gianvincenzo.zuccotti@unimi.it (G.V.Z.); 2 Department of Health Sciences, University of Milan, 20146 Milan, Italy; giulia.fiore2@studenti.unimi.it; 3 Department of Food Hygiene and Nutrition, ATS Milano Metropolitan City, 20122 Milan, Italy; a_frassinetti@yahoo.it; 4 Department of Childhood and Developmental Medicine, “Fatebenefratelli Sacco” Hospital, 20157 Milan, Italy; lisa.mariotti@asst-fbf-sacco.it; 5 Centro Cardiologico Monzino IRCCS, 20138 Milan, Italy; alberico.deltorto@cardiologicomonzino.it; 6 Department of Obstetrics and Gynecology, ASST Santi Paolo e Carlo-San Paolo University Hospital, 20142 Milan, Italy; annamaria.marconi@unimi.it; 7 Department of Biomedical and Clinical Science “L. Sacco”, University of Milan, 20157 Milan, Italy

**Keywords:** pregnancy, early nutrition, fake news, Nutripedia project

## Abstract

(1) Background. Early nutrition and lifestyle before and during pregnancy, breastfeeding, infancy, and early childhood can affect the risk of developing common non-communicable diseases during adulthood such as obesity and metabolic syndrome. To support positive long-term outcomes, it is essential to debunk fake news and provide evidence-based nutritional recommendations. “Nutripedia-Informati per Crescere” is a new tool delivering information and education on appropriate nutrition of mothers and babies during pregnancy and the first years of life. (2) Methods. Nutripedia provides the readers with evidence-based scientific contents in an easy-to-access fashion through a website, a social media page and a personalized advice app called “Nutripedia Chatbot”. (3) Results. Forty articles were published on Nutripedia website with more than 220,000 total views. Social channel activation via bloggers reached over 9 million parents. 14,698 users downloaded Nutripedia chatbot, through which a total of 1930 questions were directed to experts while over 24,000 responses were provided by the app. (4) Conclusions. The use of different communication tools delivering evidence-based nutritional information such as Nutripedia is increasing and could offer supportive strategies to provide scientific information to large audiences and contribute fighting fake news. Future research could investigate the effectiveness of this important health campaign.

## 1. Introduction

Early nutrition and lifestyle before and during pregnancy, breastfeeding and infancy, have important long-term effects on later health of the child. Particularly, this phenomenon is known as “early developmental (or metabolic) programming”, referring to the possibility to modulate early growth and metabolic pathways with potential long-term impact on adult health [1,2,3]. Indeed, many studies have consistently shown an influence of early nutrition on the risk of developing non-communicable diseases (NCDs) and other chronic diseases such as obesity, which itself is a major risk factor for NCDs [3,4].

During the late 1980s, the Developmental Origins of Health and Disease (DoHAD) hypothesis, also known as the Barker hypothesis, was developed, suggesting that there is a relationship between unfavorable fetal conditions and the development of disease in adulthood [3,5]. Later, the study of “the window of susceptibility” to nutritional programming in fetal life has highlighted fetal plasticity during embryogenic life and the possible negative impact of adverse conditions on organ and system development. The lifelong risk of NCDs is therefore influenced by a critical period, the first 1000 days of life from conception to early childhood, [4] during which most of the biological development is completed [3]. Thus, we refer to “early life” as the first stages of life for the fetus and the newborn, up until early childhood [6].

Multiple studies on famine from different geographical areas have shown that fetuses exposed to undernutrition carried a higher risk of coronary heart disease, hypertension, and metabolic syndrome later in adulthood, compared to those not exposed to starvation [6,7].

Poor maternal nutrition affects fetal development leading to irreversible changes and growth retardation. The fetus, in order to convey the little energy available to cardiac and neuronal development, carries out adaptative responses. Having developed in a poor and hostile environment, when facing a “richer” environment after birth, the child is unable to adapt, developing an increased risk of NCDs [3].

By contrast, also an obesogenic diet during pregnancy can lead fetuses to develop later in adulthood diseases such as hyperinsulinemia and hypercholesterolemia, which are related to obesity and to disease vulnerability throughout adult life [6].

Maternal undernutrition and overnutrition might act via the modulation of gene expression of newborns rather than actual gene mutations, and those changes are usually referred to as epigenetic changes [6]. When modifications in gene expression occur, also protein functioning might be altered, as the resulting newborn metabolic pathways [3,6]. These modulations of gene expression and cellular function are not fully clarified yet, however understanding epigenetic processes is fundamental since nutrition has transgenerational epigenetic effects [3]. Therefore, lifestyle, environment, and nutrition during pregnancy are epigenetic factors involved in both health and prevention of NCDs, and it is important to identify integrated life-cycle prevention interventions [3,6].

The Early Nutrition Project has investigated early life causes of obesity and its prevention strategies in a study population of 470,000 individuals. According to this study, there are three key hypotheses of metabolic programming by early nutrition and lifestyle. Firstly, intrauterine exposure to an excess of nutrients (mainly glucose) causes permanent changes of the fetus leading to obesity in postnatal life. Secondly, studies found an association between rapid weight gain in infancy and an increased risk of later obesity and adverse outcome. Lastly, children experiencing a mismatch between sub-perinatal and obesogenic childhood environments are at risk of obesity and related comorbidities [1,4].

Thus, evidence-based recommendations for an optimal early nutrition to support favorable long-term outcomes were focused on four target groups: women in preconception period, pregnant women, infants, and young children [1].

Early nutrition and lifestyle recommendations for pregnant women underly the importance of a balanced diet with an increase in dietary energy intake in late pregnancy of no more than 10% above the recommended energy intake in non-pregnant women. Moreover, requirements of several micronutrients increase to a much larger extent, therefore attention should be directed to dietary quality and the selection of foods rich in critical nutrients, including minerals and vitamins. Infants and young children recommendations point out the importance of feeding practices that aim to achieve a normal weight gain, as defined by generally accepted growth standards. Exclusive breastfeeding should be encouraged during the first 6 months of life, while during the first year of life it is important to ensure the correct introduction of complementary foods while avoiding regular cow’s milk consumption and limiting dietary sugar intake [2].

Recently, the International Federation of Gynecology and Obstetrics (FIGO) guidelines have emphasized the role of clinicians in preventing and managing women obesity before, during, and after pregnancy, harnessing the increased contact with healthcare professionals during this period [8]. Obstetricians and gynecologists are uniquely positioned to influence obesity risk and prevalence through interventions with women of reproductive age before, during, and after pregnancy. Moreover, preconception and postpartum periods are key time points for intensive nutrition and weight optimization, which may delay or prevent progression of obesity in women and reduce the trans-generational risk of transmitting it [8].

Recent studies have pointed out that prevention of NCDs starts well before conception, when adolescents are exposed to factors affecting their metabolic programming [4]. Adolescents are considered a target population for preconception prevention and healthcare providers and pediatricians should work to optimize adolescents nutrition and health [2]. It is interesting to note that the most common endocrine disorder in young reproductive-aged women, known as polycystic ovary syndrome (PCOS), is often associated with obesity and with the impairment of reproductive health.

It is recommended that adolescents with obesity and PCOS undergo a Medical Nutrition Therapy (MNT), a recommended dietary and lifestyle treatment. The approach of MNT in these patients aims at improving insulin resistance and metabolic and reproductive functions by means of personalized diets. The use of a nutrition therapy may be considered a preventive strategy to safeguard both fertility and preconception health [9].

A great collaboration with healthcare professional is needed to ensure healthy nutrition and development of children. Pediatricians can play a key role both in the education of parents and caregivers as well as in taking part to health-promoting interventions. Therefore, pediatricians can work together with other professionals in the preparation and publication of scientific articles which highlight the importance of actions focused on the concept of the first thousand days and they can contribute to support health policies and practices by spreading of scientific knowledge [10].

The available evidence strongly suggest that parents and families need to be supported in providing nurturing care with information about pregnancy and infancy to achieve the fetus and child developmental potential [11]. However, particularly in the first 1000 days of life, it is impellent to promote health nutrition and development, at the individual and collective levels [10,11].

Most new parents may feel they are not receiving enough social support and having gaps in nutritional knowledge on pregnancy and early parenthood. Pregnant women can actively seek health and nutrition information online; however, given the large amount of inaccurate information on the internet, people can easily become misinformed [12,13]. Findings suggest that approximately 60% of pregnancy-related nutrition web pages contain total or some inaccurate information [13]. Therefore, parents are likely to meet false and uncertain news concerning, above all, eating habits of mothers of children in their very delicate first 1000 days of life. This misleading and inaccurate nutrition-related information, commonly referred to as “fake news”, needs to be displaced from reliable sources by accurate and scientific evidence-based online contents to inform on healthy eating practices [13].

Online contents can be improved by simplifying access to trustworthy sources of information and asking scientists and health providers to directly contribute to online websites, making evidence-based information accessible to everyone [12,14]. Internet is also a key source of prenatal nutrition information that can lead pregnant women to achieve healthy dietary changes [14].

Preventive and promotive strategies of healthy early nutrition might include the creation of online platforms able to deliver useful information to parents, also highlighting demonstrated outcomes. [11]. Although a recent study of a web-based nutrition information program for pregnant women found no effect on neonatal outcomes, future studies need to explore women’s online engagement in modifying dietary behavior [15].

Finally, a recent randomized controlled trial investigated the effect of a mobile app as an effective intervention targeting nutrition during periconception period. As a result, high compliance and improvements in nutritional behaviors has been demonstrated and, in addition, the importance of empowering women with personalized interaction to healthy diet and lifestyle was emphasized [16]. Therefore, personalized and individualized evidence-based mobile apps represent possible interventions targeting women prior to conception, during and after pregnancy.

## 2. Aim of the Paper

This paper, introduces novel tools of e-health communication, with the scope of counteracting nutritional fake news concerning pregnancy and the first 1000 days of life. Thus, Nutripedia website was developed to spread evidence-based contents as a powerful tool against fake news and to enhance prevention through healthy early life nutrition. General population advice went along with an innovative application to deliver individualized information and intervention.

## 3. Materials and Methods

The Nutripedia project consists of two channels of communication, a hub and a mobile application, that were developed together.

Between June 2018 and November 2020 Nutripedia website and the Chatbot app were promoted in Italy via nongovernmental organization outreach, digital media sources and through scientific societies conferences. To collect quantitative and qualitative data of users’ utilization, a descriptive content analysis was conducted. We used a qualitative descriptive study to better analyze parents use of the e-health website and app during pregnancy and early life, because information about this phenomenon is scant in research literature. Thematic content analysis was used to assess users’ interactions, to understand users’ experiences and to identify main topics of interests.

The analytical software Google Analytics was embedded on the website to assess parents’ usage patterns. Extraction of numbers of online accesses, length of the web sessions, collection of number of pages visited and of most viewed pages were performed. Data collection from Chatbot app was performed thanks to the analytical processing featured in the application.

### 3.1. Nutripedia Hub

#### 3.1.1. What Is It

“Nutripedia-InformaTI per crescere “ is a parent-oriented campaign to spread up-to-date nutritional knowledge, focusing on preconception, pregnancy and early children life up to three years. Nutripedia provides the users with evidence-based scientific contents with an easy-to-access online platform. It consists of (i) a website, (ii) a social media page hosted on Facebook (FB), and (iii) “Nutripedia Chatbot”, a personalized advice app for IOS and Android.

#### 3.1.2. How Was It Born

**Figure 1:**
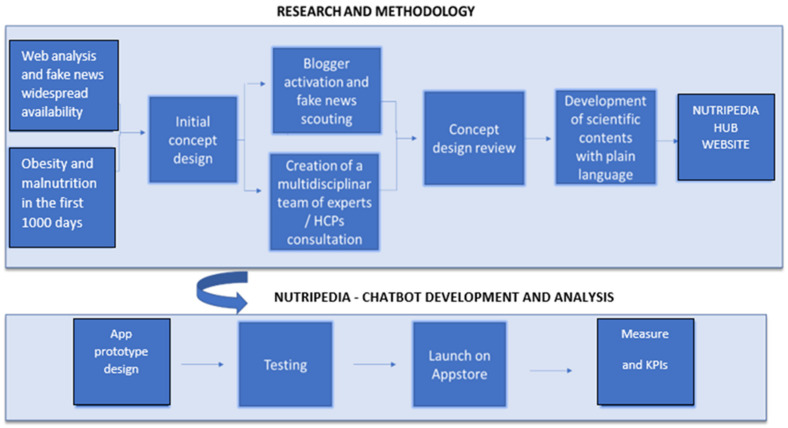
Development process and architecture of the Nutripedia website and Nutripedia Chatbot app.

Nutripedia project was developed through formative research, incorporation and performing phases (Figure 1).

The main goals of Nutripedia project are (i) the scientific dissemination of nutrition related topics, (ii) the fight against fake news spread online and among parents, providing the readers with scientific evidence (iii) sharing of blogging-parents personal experiences iv) providing personalized advice through the app.

#### 3.1.3. A New Alliance: Scientists and Bloggers (The Involved People)

A multidisciplinary team was engaged in the Nutripedia project, including a panel of health experts (the RIMMI Board) and a team of blogging-parents.

#### 3.1.4. The Scientists

The RIMMI board (Rete Internazionale Milano Materno-Infantile) is a team of experts in infant nutrition including pediatricians, dieticians, and gynecologists in Milan, Italy. In the Nutripedia campaign, the RIMMI Board oversaw:

1. Preparing scientific articles with evidence-based contents, advice, and recommendations to share and reinforce correct information online.
2. Debunking common fake news, uncertain data and recommendations circulating online and among parents, providing an up-to-date knowledge instead.
3. Delivering reliable information in an easy-to-access format to reach the large public, e.g., focusing on key information and practical advice.
4. Giving personalized advice if requested and needed.

The campaign was supported by the Danone Institute Foundation, a not-for-profit organization aiming to promote human health through developing and disseminating knowledge about the links between food and health, and to highlight the importance of nutrition in health.

#### 3.1.5. The Bloggers

Eight blogging-parents (7 mothers and 1 father) were engaged to spread the Nutripedia campaign and represent the parents’ community, including the father’s point of view. Blogging-parents were job-paid social media experts, already known to the public for their parenthood experiences. They were selected depending on number of visibility and coherence with Nutripedia project. Educated and supported by the scientific board, the virtual community of the bloggers oversaw:

1. Detecting online fake news regarding infant nutrition and identifying trends in users’ information-seeking behavior. This fake news was then corrected by the panel of experts and posted on the website in a dedicated section.
2. Share their experience in the section “la voce dei genitori-the parents’ voice” on the Nutripedia platform to promote healthy behaviors.
3. Write and publish posts on their blog about their personal experience and fake news, to encourage people to reach out the website and build a solid virtual community. Blogging parents relaunched on their blogs the contents developed by the expert team (each with 1 Instagram (IG) post and 1 FB post), with hashtags and mention of the campaign.

#### 3.1.6. The Content of the Website (Advice for General Population)

The Nutripedia website was developed through evidence-based information together with a formative research and critical analysis about nutritional information tools available online, design and performance of the page [17,18,19,20,21,22,23,24,25,26,27,28,29,30,31,32,33,34,35,36,37,38,39,40,41,42,43,44,45]. Nutripedia page was developed as a scientific support resource available online for all parents, to provide experts opinion in easy-to-access online tools. Parents habits of nutritional information searching were investigated, focusing on parameters as the time spent online, websites consultation and use of apps and social media on nutritional topics. The quality and reliability of the information were also considered.

The main fake news intercepted by bloggers were combined with timely and periodic analyses starting from queries on Google and online social media. The experts collected and incorporated these multiple data and information to set up contents and posts for the website.

**Figure 2:**
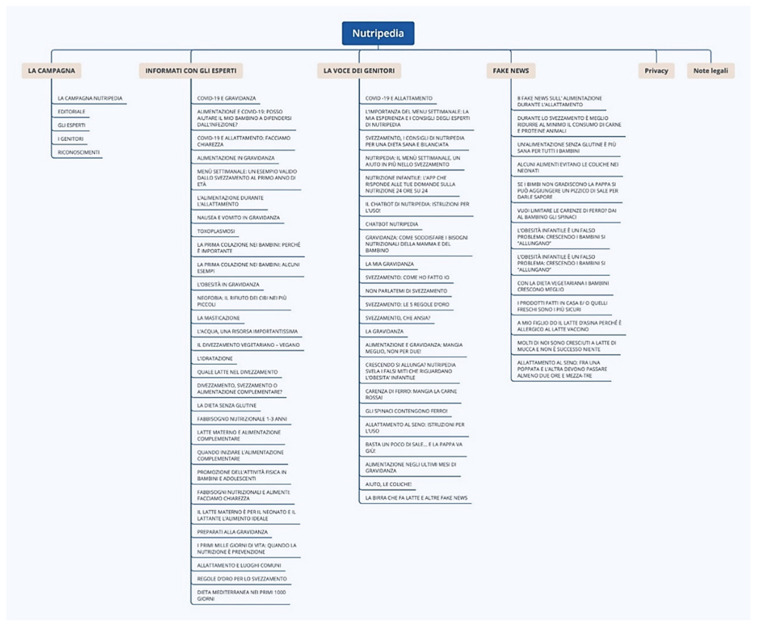
Navigation flow of Nutripedia website. The navigation flow of Nutripedia is divided into six sections “The campaign”, “The expert voices”, “The parents’ voices”, “Fake News”, “Privacy” and “Legal Information” respectively. Specific web-articles for each topic are listed in the scheme (for instance COVID-19 and pregnancy for “The expert voices” topic or Complementary feeding: my personal experience for “The parents’ voices” topic and 8 fake news about nutrition during breastfeeding for “Fake News” topic).

**Figure 3:**
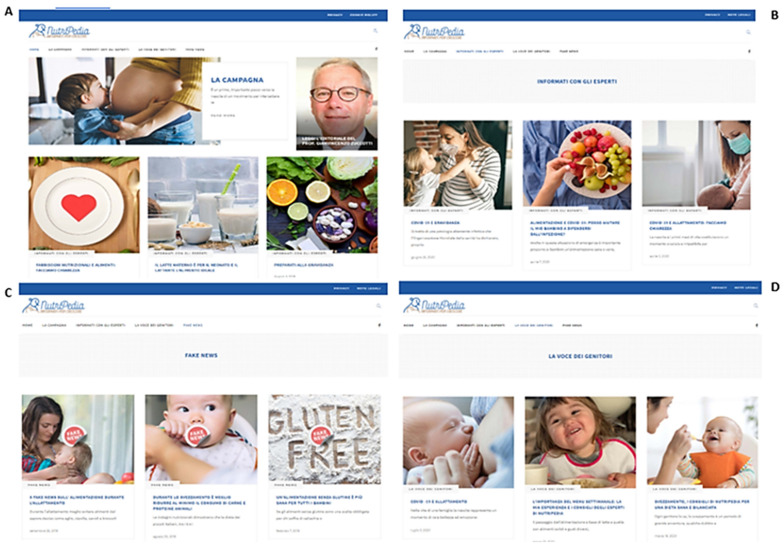
Impression on user interface (**A**), “The Expert voices ” landing page (**B**), “Fake news” landing page (**C**), “The parents’ voices ” landing page (**D**).

The navigation flow and the impression on user interface are shown in Figure 2 and Figure 3.

Contents and articles were organized into 3 categories to make them more accessible. A brief overview of the operationalization is provided in Table 1.

Contents and information were tailored specifically to meet public need. The key information was edited to be suitable for online content and website delivery.

The pillars in the design of the online contents included:

- fight against fake news, with special focus on parents’ main needs
- scientific and evidence-based approach
- engaging and easy to access format
- simple, supportive, and friendly tone.

The articles were combined with external links and additional information (e.g., National Guidelines and Recommendations) [17,18,19,20,21].

Facing the current situation about the Covid-19 pandemic, Nutripedia page evolved with specific contents. Specific pregnancy risk or uncertainty about safety breastfeeding were addressed, maintaining a scientific and evidence-based approach [17,18,19,20,21,22,23,24,25,26,27,28,29,30,31,32,33,34,35,36,37,38,39,40,41,42,43,44,45].

### 3.2. Chatbot

#### 3.2.1. What Is It

The Nutripedia-Chatbot, part of the Nutripedia project, consists of an interactive app designed to enable a direct communication between parents and health experts. The app is available for free on Apple store and Google Play store, and was developed to be up-to-date and easy to use.

The RIMMI board supported the guided conversation with users. The experts developed an outline of questions and repliesto be included in the app to form the initial checklist basis. During the initial phase, experts added 140 main topics into the chatbot with a default question and answer system, regarding nutrition from the fertile age to children first 3 years of life. Moreover, experts were engaged answering new questions posted by users.

These new topics and contents were regularly implemented into the system.

#### 3.2.2. How Was It Born (Targeted to Personalized Advice)

Nutripedia app was developed through stages of critical analysis, contents and engagement implementation, and test evaluation.

A critical analysis about online information sources and digital educational strategies was conducted. Multiple mobile technology and nutritional counselling strategies available on the web were also reviewed.

**Figure 4:**
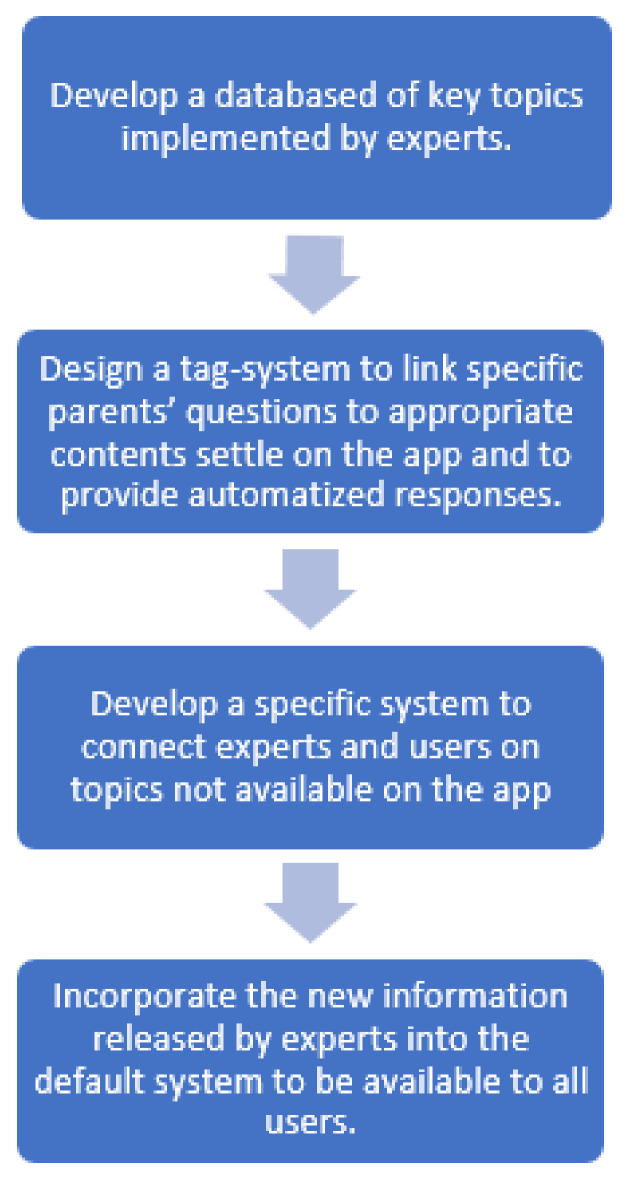
Development process of Nutripedia app.

Figure 4 shows tasks conducted processing the app.

We developed the Nutripedia-Chatbot App as a personalized digital counselling tool. The Nutripedia-Chatbot enables parents to ask specific questions about the following nutritional topics:

- Fertile age
- Pregnancy
- Breastfeeding
- Weaning
- 1–3 years
- General topic

Each entry question is evaluated against a checklist, uploaded on the system, and addressed to several topic. The pre-defined set of answers was loaded on the app based on a tag system. The tag-system was set to ensure proper response for topics and allows the user to be placed into a preset conversation about selected topic. A synonymous-word table about specific and medical terms was implemented to the database to facilitate the tag-recognition process.

**Figure 5:**
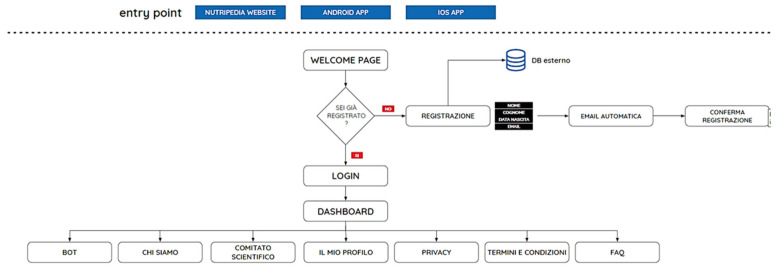
Navigation flow of the Nutripedia-Chatbot app. First impression in the welcome page is “Are you already registered?”: if NO people are asked to REGISTER with their EMAIL, if YES people are asked to LOGIN. After having logged a DASHBOARD appears with different sections: “BOT”, “About us”, “Scientific Committee”, “My profile”, “Privacy”, “Terms and Conditions” and “FAQ”.

In Figure 5 the user flow is represented.

The app was designed to find responses to the main personal uncertainties. Therefore, combined with the automized answer system, the app was settled to allow a direct connection between users and experts.

**Figure 6:**
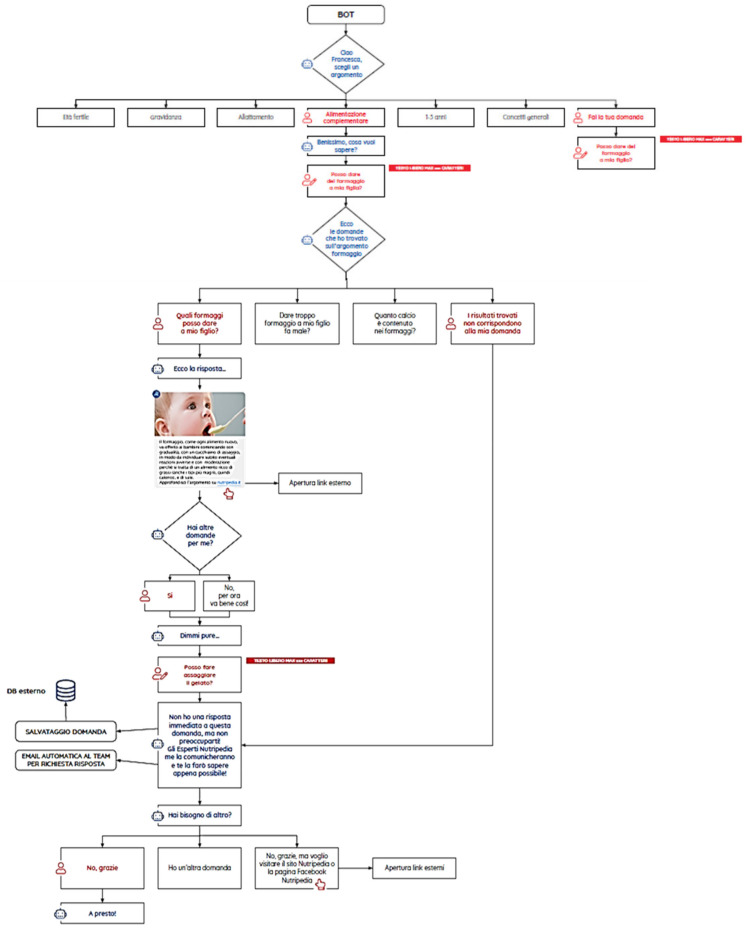
Simulation of questions functionality of the Nutripedia-Chatbot app. An example: having asked if during complementary feeding cheese is allowed for infants, the BOT presents the user 4 topics: “Which type of cheese is permitted”, “Too much cheese is dangerous?”, “Calcium content in cheeses” and “No results found”. Concerning to each topic, the BOT answer is a link to the specific article concerning to it. Following this, if BOT is asked something not registered in the app, the question is collected, and an automatic email is sent to Nutripedia Expert Team to answer it.

To meet the personalized needs of parents, questions and topics not included in the chatbot database are addressed to the experts. The app places the users into a digital and personalized conversation with experts, who will respond directly by the app as soon as possible. The chatbot uses the progressive disclosure technique where information is sequenced, and users can progressively ask further details (Figure 6).

The automated-system allows to save these new question-answer on the databased and increase the addressed contents. The app also contains a history view where delivered responses are automatically recorded and stored. This system ensures users easy access to previously requested information.

To support the reliability of responses and to provide further information external links were added. The responses were based on and supported by the main scientific documents and sources available (e.g., National Guidelines and Recommendation) [17,18,19,20,21]. Practical advice and references to component articles in the Nutripedia page were also included.

Without replacing the opinion of the pediatrician or nutritionist, the Chatbot represents a valid support that connects experts and parents and spread correct information about nutritional doubts and knowledge.

## 4. Results

### 4.1. Nutripedia Website

Forty articles regarding several scientific topics and debunking of fake news were published on the Nutripedia website with more than 220,000 total views. The most consulted articles cover breastfeeding and weaning-related topics. The main fake news interactions are related to pregnancy and early nutrition, with an average of 2000 views.

Table 2 shows the most visualized articles and fake news.

Blogging-parents wrote 23 guest posts about their experience on the dedicated website section “La voce dei genitori” (The Parents’ voice). Moreover, each blogging-parent posted a total of 10 articles about Nutripedia contents on their social blog. Relaunches on their social channels, with 1 IG post and 1 FB post, with hashtags and mentions to the campaign, were also included.

The views on the blogs were over 135 thousand (K), while over 9 million (M) users were reached through bloggers’ social channel activation: over 7.3 M users on Facebook, 1 M on Instagram and 716 K on Twitter.

More than 275,000 interaction result from Nutripedia Facebook page, with a total of 19,600 like, 1730 comments and over 2500 share posts.

Parents feedback about satisfaction, feasibility and acceptability of the website was revealed through the comments and networks on social channels and blogs. Users positively commented on the helpfulness of the website for increasing nutritional knowledge and awareness.

Some parents commented on the process being “quick” and “easy” to find information, and that using the website was preferable to other assessment methods. Parents indicated the need of easy and reliable digital tools with trustworthy information and were enthusiastic about the presence of experts against numerous fake news on the web.

The Nutripedia project has been awarded with national and international awards and acknowledgments. The campaign reached the endorsement by the Italian Association of Pediatrics (SIP) and the Italian Health Ministry and was object of media interest and national science festivals. The Nutripedia project has also been awarded and recognized internationally (Assorel Award and Positive Business Award).

### 4.2. Nutripedia Chatbot

Nutripedia-Chatbot was developed as pilot-study to perform and test digital counselling via app.

The app prototype was tested for usability and functionality issues. When testing the app, a total of 15 questions were uploaded by digital professionals and experts involved into the project. The ability of users to post their own questions, the ability of the system to provide appropriate answers using the tag-system and the feasibility of the self-implement process with the new contents addressed by the experts were evaluated.

After the up-test phase, a total of 140 default questions-answers were uploaded into the system. The contents are about nutrition from the fertile age to children’ first 3 years of life.

The App was downloaded by 14,698 users. A total of 1930 questions were directed to experts while over 24,000 responses were provided by the app, 21% of which (285) were recognized by the tag-system. Excluding repeat and test responses, 1100 responses were uploaded by experts, including 884 (80%) ad hoc responses from personal counselling. Responses directly addressed to experts were implemented as default contents to be used by new users. The parents’ main uncertainties and “compliance” doubts concerned weaning and feeding in early childhood. The main topics addressed by experts were:

- Timing of food introduction in early childhood: e.g., cheese, fruit, vegetables, fish.
- Composition of a complete meal both in complementary nutrition and after one year of age
- Foods to be preferred or avoided during pregnancy and breastfeeding
- Amount of milk during the first 6 months of life
- Problems related to breastfeeding

In Table 3 are listed the different percentage of questions to each content.

## 5. Discussion and Conclusions

Factors affecting parents’ nutritional knowledge are broad and conflicting, and include a combination of online tools, social environment, peers’ advice, medical assistance, and personal convictions. These multiple sources of information can negatively affect parents’ knowledge mobilization contributing to dissemination of uncertain and incorrect advice and information.

The use of digital apps in health promotion interventions is emerging and could serve as a supportive strategy, with greater population reach, to provide scientific information and to fight fake news.

Nutripedia is a mobile campaign developed specifically to promote correct information for general population (Nutripedia website) and to address the individual doubts and questions from parents (Nutripedia app). Website contents and information are all evidence-based, aiming at providing knowledge to parents which is reliable and recognized by the scientific community [17,18,19,20,21,22,23,24,25,26,27,28,29,30,31,32,33,34,35,36,37,38,39,40,41,42,43,44,45].

Some topics were investigated more commonly. This may suggest uncertainty about specific topics in parents and emphasize the importance of having a reliable source of information. Moreover, different users addressed to the experts similar personalized questions or concerns on the same topics, which reinforces the suitability of the self-implementation provided by the application. Hence the importance of the Nutripedia-Chatbot application that, by providing correct information to families, allows a real-time “more personalized intervention” based on individual questions from parents while the website responds to the needs of a wider population.

As a pilot study, the purpose was to create and test the Nutripedia application and to examine its feasibility to personalized advice. The Nutripedia-Chatbot app has been implemented and supported by only 4 RIMMI experts for 2 years. Despite the end of test-evaluation phase, the app still provides specific answers ensured by the tag-system and self-implementation process. A large users’ interaction ensured to cover multiple nutrition topics related to early parenthood.

Because the project’s principal goal was the proof-of-concept exploratory phase of the newly developed application, follow-up data have not been generated. Furthermore, users’ feedback regarding their perception of the application’s reliability and contribution to meet personal doubts was not collected, as well as knowledge improvement and change of user behavior before and after using Nutripedia. This is a limit of the project.

Nevertheless, the aim of this proof-of-concept study was to examine the applications basic concept as an initial research stage. However, this first experience represents a first step to understand the potential effectiveness of the Nutripedia educational campaign by identifying possible critical aspects that can be improved. It also provides elements to evaluate the possible extension and replicability of the campaign to develop a recognized and available tool at national level. Without replacing the clinical interaction between parents and pediatricians, Nutripedia could be a supportive tool for health-care professionals work by providing nutritional advice and real-time support for parents. As these new tools are inexpensive yet could generate a high value in terms of NCDs prevention and cost reduction, policy makers should embrace these instruments to provide appropriate evidence-based scientific information to the general population, encouraging healthy lifestyle changes and dietary patterns. 

Future research could possibly investigate the effectiveness of the campaign using different indicators, as changes in parental attitudes and behavioral intentions or usability and reliability by pediatricians.

The project could also be performed at a larger scale, thanks to stronger promotional campaign and the support of more experts in providing answers to parents’ users through the chatbot app.

Besides, the usefulness of the various information channels could also be assessed with a rating scale or a questionnaire by users with the possibility of proposing suggestions for better communication.

However, in addition to mainly quantitative survey methods, qualitative methods such as in-depth interviews and focus groups involving children’s parents and pediatricians could provide useful information to improve the efficacy and feasibility of the campaign.

Finally, it should be considered that unhealthy nutritional choices by pregnant women can be dictated by lack of information, but also by lack of economic resources and time. This should be addressed as a serious drawback. Information is not the only issue in improving maternal and fetal outcomes.

## Figures and Tables

**Table 1:** Overview of the website content and short description of the module.

Website Module	Short Description of the Module
Informati con gli esperti (*The Expert voices*)	Scientific, evidence-based information expanded with articles and contents delivered by the experts and focused on the main nutritional topics related to pregnancy, breastfeeding, first 1000 days of life, and early parenthood. Practical strategies and solutions, advice and example were also included (e.g., foods for adequate infant breakfast or weaning menus).
La voce dei genitori (*The parents’ voices*)	Blogging-parents opinions and negative experiences with incorrect information and fake news. A cooperation between blogging-parents and the project as a strategy to address and motivate parents at meeting reliable information and adopt healthy behaviors.
Fake news	Debunk and scientific implementation of the most commonly circulating fake news. Dedicated section “True or False?”.

**Table 2:** The main articles and the main fake news visualized on the page.

Informati Con Gli Esperti (The Expert Voices)	Topics	Views	Fake News	Topics	Views
Example of weekly menu from weaning to first years	Weaning	6987	Avoid carbs to weight up in the last trimester of pregnancy	Pregnancy	2124
Mediterranean diet in the first 1000 days of life	General	5730	Homemade food and fresh vegetables are safer	Weaning	1745
Best weaning practices	Weaning	4933	Top 8 fake news about breastfeeding (ex. Eat for two or avoid specific foods such onions or garlic)	Breastfeeding	1531
Breastfeeding advice	Breastfeeding	4571	Specific food ensures to avoid infant colic	Weaning	1451
Nutrition during breastfeeding	Breastfeeding	2719	Many of us grew up on cow’s milk and nothing happened	Breastfeeding	1292

**Table 3:** Percentage of question to each content.

Item	%
Fertile age	3.6
Pregnancy	18.8
Breastfeeding	18.5
Weaning	28.3
1–3 years	22.3
General topic	8.5
